# Knowledge, Practices, and Attitudes of Emergency Contraception among Female University Students in KwaZulu-Natal, South Africa

**DOI:** 10.1371/journal.pone.0046346

**Published:** 2012-09-26

**Authors:** Muhammad Ehsanul Hoque, Shanaz Ghuman

**Affiliations:** 1 Department of Public Health, School of Health Care Sciences, University of Limpopo (Medunsa Campus), Pretoria, South Africa; 2 Department of Environmental Health, Mangosuthu University of Technology, Durban, South Africa; IPO, Inst Port Oncology, Portugal

## Abstract

**Objective:**

The purpose of this study is to investigate the knowledge, practices, and attitudes among female university students in South Africa regarding emergency contraceptives (EC).

**Methods:**

A cross-sectional study was conducted among 582 female university students who were selected using multi-stage sampling techniques. Multivariate logistic regression analysis was used to find significant predictors for EC awareness.

**Results:**

The average age of the female students was 20.9 years (SD = 3.0) and 57.2% were presently sexually active. Overall, 49.8% of the participants reported having heard about EC prior to the study. Regarding sexual activities among the female students, 53.2% reported to have sex, and 21.2% of the sexually experienced students used EC prior to the study. Regarding the effectiveness of EC, 29.5% students said it could be used up to 72 hours after unprotected sexual intercourse, and 8% said it could be used just before sex. About two-thirds (61.8%) would recommend the use of EC and 63.2% would use it if they needed. The multivariate analysis indicated that students who were older (>20 years), presently sexually active, and living with their parents were more likely to be aware of EC (p<0.05).

**Conclusion:**

The students’ knowledge and utilization of EC were low. Health education and promotion should be targeted towards these students, and the EC services should be offered on campus.

## Introduction

Emergency Contraception (EC) refers to a group of birth control contraceptive modalities. If it is used following unprotected sexual intercourse within a defined time period, an unwanted pregnancy could be prevented [1]. For example, EC can reduce the risk of an unintended pregnancy after unprotected sexual intercourse or contraceptive failure by at least 75% to 99%, if taken within 72 hours of sexual intercourse [2], [3]. A recent research suggests that combined EC pills are moderately effective even if it was started between the third and fifth day, up to a maximum of 120 hours following the act of sexual intercourse [4], [5].

EC is largely underutilized around the world. It has been referred to as one of the best kept “secrets” in Reproductive Health [6]. In many low income countries, the lack of knowledge about and the access to EC has resulted in women resorting to unsafe or illegal abortions. Every year, unplanned pregnancies have led to at least 50 million abortions worldwide, many of them being unsafe and subsequently resulting, in approximately 80 000 maternal deaths. This contributes significantly to maternal morbidity and mortality [7]. Knowledge and practice on emergency contraception are particularly important as a result of the high rates of unwanted and teenage pregnancies and soaring STI’s and HIV/AIDS rates. Different studies, however, have shown that the knowledge and practices in relation to emergency contraception are limited amongst the female university students [8]–[12].

In South Africa (SA), teenage pregnancies occurrs from unstable relationship and they are usually unplanned or unwanted pregnancies. In fact, in 2004, 13% of abortions conducted at government health institutions were for women younger than 18 years of age, which was considerably higher than their contribution to the total fertility rate [13]. Although several contraceptive methods, including EC, are available and free to users at all public sector health facilities across the country, however high rates of teenage and unintended pregnancies still persists in South Africa. It is estimated that up to 75% of pregnancies in South Africa are unintended, with the highest proportion being among adolescents, and with a very high incidence of sexual assault [14]–[16].

**Table 1:** Demographic information’s and their association with awareness of EC^a^.

Variable	Not heard of EC	Heard of EC	Chi-square value	p-value
**Age (in years)**				
<20	163 (38.08)	102 (24.06)	20.49	<0.001
20–25	244 (57.01)	303 (71.46)		
>25	21 (4.91)	19 (4.48)		
Mean age (SD)	20.9 year (3.0)			
**Matriculated from**				
Rural School	240 (56.07)	253 (59.67)	1.13	0.288
Urban School	188 (43.93)	171 (40.33)		
**Marital status**				
Single	393 (91.82)	397 (93.63)	1.03	0.309
Married/living together	35 (8.18)	27 (6.37)		
**Currently living with**				
Parents	84 (19.63)	107 (25.24)	14.10	0.007
Hostel	261 (60.98)	262 (61.79)		
Renting with friends	50 (11.68)	34 (8.02)		
Boyfriend	19 (4.44)	5 (1.18)		
Other	14 (3.27)	16 (3.77)		
**Year of study at the university**				
First	145 (33.88)	108 (25.47)	16.46	<0.001
Second	211 (49.30)	199 (46.93)		
Third	72 (16.82)	117 (27.59)		

a Values are given as number and relative frequencies.

In order to increase the public health benefits, of widespread hormonal EC availability, potential users must be well informed about the use of EC. Specifically, women in their child-bearing age must know that EC does exists, know the time limits within which EC may be effective, and know where EC can be obtained from quickly. Without this knowledge, women will miss the opportunity to access free EC at public sector health facilities, where 84% of contraceptive users, in South Africa obtain their contraception [17]. Women’s lack of information and knowledge about how to protect themselves from pregnancy and prevent unplanned pregnancies can be very risky and disastrous. Given the importance of EC, the purpose of this study are to investigate the knowledge, practices, and attitudes among female university students in South Africa regarding EC.

## Materials and Methods

### Study Setting

The Mangosuthu University of Technology (MUT) is situated about 18 kilometers, south of the busiest port city (Durban) of South Africa in KwaZulu-Natal (KZN) province. The programmes are prioritized to serve mainly, the students from the historically, disadvantaged communities. In 2011, the student population for the day students was 8345, of which 4244 were females and 4101 were males. MUT offers postgraduate qualifications and national diplomas, with academic teaching continuing during both the day and the evenings. There are 3 Faculties, namely, Natural Sciences, Engineering and Management Sciences as well as an Extended Curriculum Programme.

**Table 2:** Sexual behavior, contraceptive uses and pregnancy status of the university students’^a^.

Variable	Not heard of EC	Heard of EC	Chi-square value	p-value
**Ever had sex**				
No	244 (57.01)	155 (36.56)	35.78	<0.001
Yes	184 (42.99)	269 (63.44)		
Median age at first sex (Range)	19 years (14–25)			
**Presently sexually active (n = 453)**				
No	90 (48.91)	104 (38.66)	4.69	0.030
Yes	94 (51.09)	165 (61.34)		
**Ever used contraceptive (n = 453)**				
No	62 (33.70)	100 (37.17)	0.58	0.448
Yes	122 (66.30)	169 (62.83)		
**Types of contraceptive used (self or partner) (n = 453)**				
OCP	58 (31.52)	69 (25.65)	7.85	0.097
Injectable	47 (25.54)	70 (26.02)		
Female condoms	8 (4.35)	15 (5.58)		
IUD	0 (0.00)	9 (3.35)		
Others	71 (38.59)	106 (39.41)		
**Used contraceptive in the last intercourse (self or partner) (n = 453)**				
No	79 (42.93)	110 (40.89)	0.187	0.665
Yes	105 (57.07)	159 (59.11)		
**Ever been pregnant**				
No	144 (78.26)	219 (81.41)	0.682	0.409
Yes	40 (21.74)	50 (18.59)		
**Had unwanted pregnancy**				
No	21 (52.50)	23 (46.00)	0.376	0.540
Yes	19 (47.50)	27 (54.00)		
**Had induced abortion**				
No	38 (95.00)	32 (64.00)	12.36	<0.001
Yes	2 (5.00)	18 (34.00)		

a Values are given as number and relative frequencies.

### Study Design, and Data Collection

This was a descriptive, cross-sectional study conducted in September 2011 among full–time undergraduate MUT students. The Multi-stage sampling techniques were used to approach the participants. The study was conducted by means of a questionnaire survey. The questionnaire included questions on demographic variables, sexual behavior, knowledge, practices and attitudes regarding EC. The data collection procedure has been explained elsewhere [18].

**Table 3:** Knowledge of students who ever heard about EC.

Variables	Frequency	Percentage
**Main source of information**		
Media	83	9.7
Doctors/Nurses	104	24.5
CHWs	128	30.2
Friends	234	55.2
Relatives	77	18.2
Book or Magazine	97	22.9
**Which one of these can be used for EC?**		
Norlevo	157	37.0
Microval	88	20.8
Ovral	115	27.1
Nordette	34	8.0
IUCD	12	2.8
IUD	37	8.7
**From where can you access EC?**		
Public health facilities	299	70.5
Private doctors/Clinics	148	34.9
Private hospitals	121	28.5
Pharmacists	261	61.6
**Can you get EC without a prescription?**		
No	94	22.2
Yes	129	30.4
Do not know	201	47.4
**When EC can be used effectively?**		
Just before sex	34	8.0
Within 24 hrs after unprotected sex	86	20.3
Within 72 hrs after unprotected sex	125	29.5
More than 72 hrs but less than 120 hrs after unprotected sex	44	10.4
Until the next menstrual period	4	0.9
Even after a missed period	5	1.2
Don’t know	126	29.7
**Main adverse effects of EC**		
Infertility	148	34.9
Irregular menses	89	21.0
Bleeding	109	25.7
Nausea and vomiting	39	9.2
Weight gain and pain	26	6.1
Death	13	3.1

### Ethical Considerations

Ethical permission for the study was obtained from the Ethics task team of the Faculty of Natural Sciences Research and the Publications Committee of MUT. Written informed consent of the participants was obtained. Confidentiality of the participants was maintained at all times. To further maintain anonymity, no forms of identifiers were in the questionnaires, as code numbers were used. Participation was voluntary and the participants were informed that they could withdraw from the study at any stage of the interview, if they so desired, without any penalty or explanation.

**Table 4:** Utilization and attitudes towards EC among the students.

Variables	Frequency	Percentage
**Ever used EC**		
No	334	78.8
Yes	90	21.2
**Source of EC at the last time**		
Public Health Facility	52	57.8
Private doctor/clinic	10	11.1
Private hospital	2	2.2
Pharmacist	22	24.4
District surgeon for rape	4	4.4
**Will you recommend EC**		
No	162	38.2
Yes	262	61.8
**Which circumstances you** **will recommend EC**		
Rape	106	40.5
Unprotected sex	27	10.3
Contraceptive mishap	11	4.2
All of the above	113	43.1
Do not know	5	1.9
**Should EC be included in** **orientation program**		
No	36	8.5
Yes	298	70.3
Do not know	90	21.2
**Should EC be provided on** **Campus**		
No	130	30.7
Yes	294	69.3
**Would you use EC in future**		
No	156	36.8
Yes	268	63.2

### Data Analysis

Data were entered into Microsoft excel 2003 spreadsheet and imported to SPSS 17.0.1 for window version for analysis. Both bivariate and multivariate analysis techniques were applied, to identify the factors associated with the likelihood of being aware of EC. The Chi-square test was used to test the association. The variables that were significant in the bivariate analysis were re-examined using stepwise binary logistic regression, in order to identify the significant predictors after controlling other variables. P-values were reported to three decimal places, with values less than 0.001, reported as <0.001 and values less than 0.05 were considered statistically significant.

**Table 5:** Results of multivariate logistic regression modelling to determine factors independently associated with awareness of EC among female university students.

Variables in the Equation^a^	B	S.E.	Wald		p-value	Odds Ratio (OR)	95% C.I. OR	
							Lower	Upper
**Age group** b								
20–25 years	1.158	0.252		21.043	0.000	3.183	1.941	5.221
>25 years	1.093	0.453		5.820	0.016	2.982	1.227	7.244
Presently sexually active	0.732	0.213		11.849	0.001	2.078	1.370	3.152
Currently living with^c^								
Parents	1.207	0.557		4.706	0.030	3.345	1.124	9.959
In university residence	0.063	0.474		0.018	0.894	1.065	0.421	2.694
Renting with friends	−0.181	0.538		0.114	0.736	0.834	0.291	2.392
Boyfriend	−2.012	0.705		8.135	0.004	0.134	0.034	0.533
Constant	−0.996	0.535		3.468	0.063	0.369		

a Variable(s) entered on step 1: age group, Matriculated, Marital status, Year of study, presently sexually active, ever used contraceptive, Ever been pregnant, and currently living with.

b <20 years as reference group,

c Others as reference group.

## Results

A total of 852 students completed the questionnaires. Table 1 summarizes the demographic information, as well as their association with awareness of EC. The average age of the female students was 20.9 years (SD = 3.0). The majority of the students were between the ages of 18 and 24 years old (90.1%) and single (92.7%). More than half (61.4%) were staying in the university hostels, and almost half (48.1%) were in their second year of university studies. Students over the age of 20 years, were more likely to have heard of EC, than their younger counterparts (p<0.001). Student who they were staying with was significantly associated with awareness of EC (p = 0.007).

Participants’ sexual behavior and contraceptive uses have been analyzed (Table 2). Regarding sexual activities among the female students, 53.2% reported to have sex prior to the study and the median age of first sexual encounter was 19 years. More than half (57.2%) were presently sexually active. Amongst those who ever had sex, about two-thirds (64.2%) ever used contraceptives, and 58.3% used it during their last sexual intercourse. The main methods of contraceptives used by the students were oral contraceptive pills (28.0%) followed by injectables (25.8%). One in five female students reported being pregnant (19.9%) of which 51.1% were unwanted and over a fifth (22.2%) had induced abortions. Students who had sex, as well as those who are presently sexually active, were more likely to have heard of EC than their counterparts (p<0.001, p = 0.030 respectively). Also, students having induced abortion had a higher chance of knowing about EC (p<0.001).

The questionnaire specifically asked about EC (Table 3). Overall, 49.8% of the participants reported of having heard about EC prior to the study. Very little information was provided by the media and HCW’s, but friends were the source of information for 55.2% of the female students, who had heard about EC. Regarding accessing EC, more than two-thirds (70.5%) knew that EC is available at public health facilities and 61.6% indicated from pharmacists. Less than a third (30.4%) knew that a prescription is not required to obtain EC from pharmacists. When asked to choose a method from a list of practices and products that have been used for EC, the most commonly chosen methods were oral contraceptives (Norlevo, Ovral, Microval, Nordette). With regard, to when EC could be used effectively, 29.5% students said it could be used up to 72 hours after unprotected sexual intercourse, and 8% said it could be used just before sex. The major adverse effects that the students linked with EC were infertility and bleeding (34.9% and 25.7%) respectively.

The participants’ EC utilization and attitudes have been analyzed (Table 4). More than a fifth (21.2%) of the sexually experienced students used EC, and amongst them 57.8% obtained EC from the public health facility, during their last use. The students who knew about EC, 61.8% would recommend its use, and amongst them 43.1% mentioned they will recommend it in case of rape, unprotected sex or contraceptive mishaps. More than two-thirds (70%) of the respondents called for information about EC in the orientation program, as well as that EC should be provided at the university health clinic. If EC is needed, however 63.2% of the students mentioned of using EC in future.

Backward logistic regression analysis was carried out to find the significant predictors for awareness of EC. Initially, in the model, all the variables that had significant association with awareness of EC, were included in the model (Table 5). The variables were then removed, one by one from the model, which did not have a significant impact on the model. The results indicated that the students who were between the ages of 20–25 years, were three times more likely to be aware of EC, than those who were below the age of 20 years (OR = 3.2, p<0.001). Presently sexually active students were twice more likely to be aware of EC, than non-sexually active students (OR = 2.1, p = 0.016). Lastly, students living with parents had three times more chances of knowing about EC, than those living in other places (OR = 3.3, p = 0.030).

## Discussion

This study investigated female university students’ knowledge, practices, and attitudes towards EC. The findings of this study indicate that knowledge, and practices of EC were relatively low but had positive attitudes regarding EC among the female university students.

The majority of the students were single and between the ages of 20 and 25 years. More than half (53.2%) reported to have sex prior to the study, and among them 35.8% never used any contraceptive methods. More than half (51.1%) of the pregnancies were unwanted and 22.2% had induced abortions. These findings indicate that university students are at risk of unsafe abortions, with possible consequences of genital infection and infertility [10]. A study from Ghana also reported similar findings [19].

Overall, 49.8% of the participants reported having heard about EC prior to the study, which is lower than the previous study (56.5%) conducted amongst other tertiary students of the very same region [20]. Many other studies conducted among university students found higher rates. For example, in Cameroon (63%), Ghana (51.4%), Nigeria (67.8%), Nepal (66%), Mexico (95%) and in the USA (94%) [15], [19], [21]–[24]. A South African study conducted among young women aged 15–24 years attending public sector health facilities’ reported that only 17% of the women had ever heard of emergency contraception [25]. In this study, 50.2% never heard of EC. Such findings highlight the need for wider education on reproductive health in general and especially of EC.

A large number of students heard about EC from their friends (55.2%) and a few had heard it from HCW’s. This finding is in line with other South African studies [20], [26]. These findings suggest that peer education approaches might be helpful in increasing EC awareness. A study conducted among pharmacists and doctors regarding EC, reported that they were reasonably informed of the methods, and there is a need to improve their knowledge [27]. HCW’s need to be educated about methods, safety, time limits and side effects of EC.

The present study reveals a lack of knowledge about EC among the participants. More than two-thirds (69.6%) did not know that a prescription is not required to obtain EC from pharmacists, and 29.7% did not know the right time limits for it’s effectiveness. Similar results have been reported from other African universities as well as from South Africa [8], [9], [20], [26]. Since the majority of the students’ source of information was peer-related, it is therefore not surprising to see that the correct knowledge of EC, and its timing was very poor among those who knew about it [28].

The utilization of EC among the sexually active students was relatively low (21.2%) compared to 28% in another South African study [29]. A South African study conducted among sexually active women attending public health clinics reported that 13% of the women ever used EC [26]. The EC utilization rate was higher than that of other African campuses. Studies reported 7.4% in Cameroon, 5.7% in Nigeria, 4.9% in Ethiopia [10]–[12]. The lack of knowledge of its use and side effects, concerns associated with cultural and societal beliefs, and misconceptions about its utilization could be the reasons for the low usages [29].

Most of the female students had a positive attitude towards the utilization of EC, as they would use it in future if required, and would recommend it to others. They also indicated that EC’s information’s should be included in the university orientation program. This is in line with Ghanaian students, who also had similar attitudes [19]. This indicates that the students are interested in learning more about EC, and that the university management should address this issue.

### Study Limitations

There might be some methodological and selection biases. The sample was quite large and representative, and thus minimized sample bias. The study population consisted of students at one university, thus results may not be generalizable to other universities. Because of the sensitive nature of the study and self-reporting, information bias could be introduced. To minimize this bias, anonymity and confidentiality were maintained.

### Conclusion

There was a low level of awareness and practices regarding EC among the female university students. The majority of the students had a positive attitude towards using EC in future if they had to. Thus, there is an urgent need to educate the university students about EC. Carefully designed education programs and the promotion of EC in the existing student health clinic on campuses, as well as discussions during the orientation programs need to address the issues of unwanted pregnancies and sexually transmitted diseases.
